# The *RAD52* S346X variant reduces risk of developing breast cancer in carriers of pathogenic germline *BRCA2* mutations

**DOI:** 10.1002/1878-0261.12665

**Published:** 2020-04-25

**Authors:** Aaron W. Adamson, Yuan Chun Ding, Carlos Mendez‐Dorantes, Adam M. Bailis, Jeremy M. Stark, Susan L. Neuhausen

**Affiliations:** ^1^ Department of Population Sciences Beckman Research Institute of City of Hope Duarte CA USA; ^2^ Department of Cancer Genetics and Epigenetics Beckman Research Institute of City of Hope Duarte CA USA; ^3^ College of Health Professions Thomas Jefferson University Philadelphia PA USA

**Keywords:** BRCA2, breast cancer, double‐strand break repair, RAD52

## Abstract

Women who carry pathogenic mutations in *BRCA1* and *BRCA2* have a lifetime risk of developing breast cancer of up to 80%. However, risk estimates vary in part due to genetic modifiers. We investigated the association of the *RAD52* S346X variant as a modifier of the risk of developing breast and ovarian cancers in *BRCA1* and *BRCA2* mutation carriers from the Consortium of Investigators of Modifiers of BRCA1/2. The *RAD52* S346X allele was associated with a reduced risk of developing breast cancer in *BRCA2* carriers [per‐allele hazard ratio (HR) = 0.69, 95% confidence interval (CI) 0.56–0.86; *P* = 0.0008] and to a lesser extent in *BRCA1* carriers (per‐allele HR = 0.78, 95% CI 0.64–0.97, *P* = 0.02). We examined how this variant affected DNA repair. Using a reporter system that measures repair of DNA double‐strand breaks (DSBs) by single‐strand annealing (SSA), expression of hRAD52 suppressed the loss of this repair in *Rad52^−/−^* mouse embryonic stem cells. When hRAD52 S346X was expressed in these cells, there was a significantly reduced frequency of SSA. Interestingly, expression of hRAD52 S346X also reduced the stimulation of SSA observed upon depletion of BRCA2, demonstrating the reciprocal roles for RAD52 and BRCA2 in the control of DSB repair by SSA. From an immunofluorescence analysis, we observed little nuclear localization of the mutant protein as compared to the wild‐type; it is likely that the reduced nuclear levels of RAD52 S346X explain the diminished DSB repair by SSA. Altogether, we identified a genetic modifier that protects against breast cancer in women who carry pathogenic mutations in *BRCA2* (*P* = 0.0008) and to a lesser extent *BRCA1* (*P* = 0.02). This *RAD52* mutation causes a reduction in DSB repair by SSA, suggesting that defects in RAD52‐dependent DSB repair are linked to reduced tumor risk in *BRCA2*‐mutation carriers.

## Graphical abstract

We report that the *hRAD52 S*346X variant reduces risk of developing breast cancer in individuals carrying germline *BRCA2 *pathogenic variants. The reduced frequency of single‐strand annealing with this *RAD52* variant resulted in a reduction in DNA double‐strand break repair. We observed reduced nuclear levels of RAD52 S346X, potentially explaining the reduced frequency of single‐strand annealing.

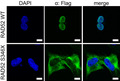

**Abbreviations**
CI: confidence interval
CIMBA: Consortium of Investigators of Modifiers of BRCA1/2
DSB: DNA double‐strand break
GFP: green fluorescent protein
HDR: homology‐directed repair
HR: hazard ratio
MAF: minor allele frequency
mESCs: mouse embryonic stem cells
NLS: nuclear localization sequence
PARP: poly(ADP‐ribose)polymerase
RMD: repeat‐mediated deletion
sgRNAs: single‐guide RNAs
SSA: single‐strand annealing
ssDNA: single‐stranded DNA
WT: wild‐type

## 1 Introduction

The human DNA repair protein, RAD52 (hRAD52), is an important factor in several different aspects of genome maintenance (Jalan *et al.*, 2019). One of the best‐defined roles of hRAD52 is as a key mediator of DNA double‐strand break (DSB) repair by single‐strand annealing (SSA) (Mendez‐Dorantes *et al.*, 2018; Stark *et al.*, 2004). SSA proceeds through the annealing and ligating together of complementary single strands of repetitive genomic sequences (repeats) flanking a DSB, providing a mechanism of DSB repair that does not conserve genome structure. SSA has been implicated in joining repeats separated by > 20 kbp, resulting in very large deletions of chromosomal DNA (Mendez‐Dorantes *et al.*, 2018). The importance of hRAD52 in SSA is likely due to its various biochemical activities, including binding single‐stranded DNA (ssDNA) and stabilizing double‐stranded DNA (dsDNA) formed upon hybridization of complementary ssDNA (Brouwer *et al.*, 2017). Importantly, while hRAD52 plays a major role in SSA, it is not part of the central apparatus controlling the repair of DSBs by homology‐directed repair (HDR) (Ceccaldi *et al.*, 2016). Loss of multiple mechanisms of DSB repair may explain the synthetic lethality observed upon depletion of RAD52 in cells with hypomorphic *BRCA2* mutations (Feng *et al.*, 2011).

**Fig. 1:**
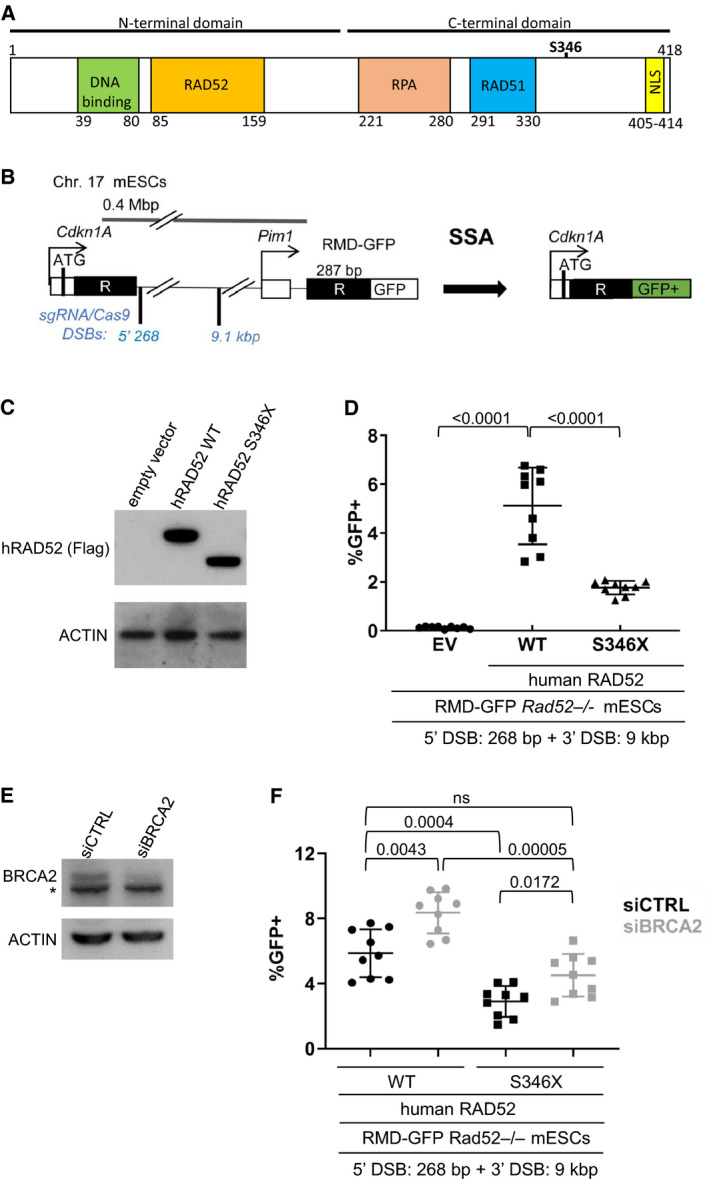
RAD52 S346X is dysfunctional in promoting SSA. (A) The domain map of human RAD52. The N‐terminal domain (1–212) contains DNA binding and self‐association regions. The C‐terminal domain (213–418) contains RPA and RAD51 interacting regions and a nuclear localization signal (NLS). (B) Diagram of the RMD‐GFP SSA reporter. Two tandem repeat (R) sequences are separated by 0.4 Mb and positioned such that SSA generates a *Cdkn1a*‐GFP fusion gene. SSA is induced by two DSBs between the repeats, with one DSB downstream from the 5′ repeat (5’268) and a second DSB 9.1 kbp upstream of the 3′ repeat. Shown are the single‐guide RNA (sgRNA)/Cas9 targeting sites for each DSB. Not to scale. (C) Flag immunoblot showing expression Flag‐hRAD52 WT and Flag‐hRAD52 S346X in RMD‐GFP *Rad52^−/−^* mESCs. (D) hRAD52 S346X is able to promote SSA but with a > 2‐fold decrease as compared to hRAD52 WT. *Rad52^−/−^* mESCs with RMD‐GFP were transfected with the 268 bp and 9 kbp sgRNA/Cas9 expression vectors along with a control EV, Flag‐hRAD52, or Flag‐hRAD52 S346X complementation vectors. Shown is the percentage of GFP^+^ cells from this experiment, normalized to transfection efficiency. *n* = 9. Lines represent the mean with SD. The numerical comparisons represent the *P*‐value determined using multiple *t*‐test with Holm–Sidak correction. (E) BRCA2 immunoblot showing depletion of BRCA2 in RMD‐GFP *Rad52^−/−^* mESCs, transfected with a pool of four BRCA2 siRNAs (siBRCA2). (∗) Nonspecific band. (F) Depletion of BRCA2 causes an increase in the ability of hRAD52 WT to promote SSA. RMD‐GFP *Rad52^−/−^* mESCs were transfected with the 268 bp and 9 kbp sgRNA/Cas9 expression vectors, either Flag‐hRAD52 WT or Flag‐hRAD52 S346X complementation vectors, along with a nontargeting siRNA (siCTRL) or siBRCA2. Shown is the percentage of GFP^+^ cells from this experiment, normalized to transfection efficiency. *n* = 9. Lines represent the mean with SD. The numbers shown above each comparison represent the *P*‐value determined using multiple t‐test with Holm–Sidak correction. (ns) Not significant.

Because hRAD52 and BRCA2 play distinct roles in DSB repair, we examined the ability of the *RAD52* S346X truncation variant (Fig. 1A) to act as a modifier of susceptibility to breast and ovarian cancers in *BRCA1* and *BRCA2* mutation carriers. Accordingly, we tested the association of *RAD52* S346X with risk of developing breast or ovarian cancer in a large cohort of *BRCA1* and *BRCA2* mutation carriers from the Consortium of Investigators of Modifiers of BRCA1/2 (CIMBA) (Chenevix‐Trench *et al.*, 2007). Based on the cellular function of hRAD52 in DNA repair, we tested the effect of *RAD52* S346X on repair of DSBs (Mendez‐Dorantes *et al.*, 2018) by SSA in conditions of both normal and reduced levels of BRCA2.

## 2 Materials and methods

### 2.1 Association between *RAD52* S346X and risk of developing breast and ovarian cancers in carriers of pathogenic *BRCA1* and *BRCA2* mutations

We initially identified the *RAD52* S346X variant (NM_134424.3:c.1037C>A, rs4987207) in an African‐American breast cancer case while testing for mutations in DNA damage response genes (Fig. S1A). This variant was sufficiently common [minor allele frequency (MAF) of 0.017 in the ExAC database (Lek *et al.*, 2016)] that we could investigate its association with cancer in *BRCA1* and *BRCA2* mutation carriers. In order to assess whether this mutation modified the risk of developing breast or ovarian cancer in women carrying pathogenic *BRCA1* or *BRCA2* mutations, we nominated this variant to the OncoArray project (Amos *et al.*, 2017) for genotyping the *BRCA1/2* mutation carriers in CIMBA (Chenevix‐Trench *et al.*, 2007). All participants in CIMBA had been previously enrolled and consented to studies through their respective institutional review board‐approved protocols. The OncoArray also was run on samples in the Breast Cancer Association Consortium (BCAC) so we looked in the BCAC summary data for this specific SNP.

The association analysis of the *RAD52* S346X variant with breast or ovarian cancer risk was carried out within a survival‐analysis framework. The time‐to‐event phenotype for each individual was defined by age at breast or ovarian cancer diagnosis or age at last follow‐up as described previously (Ding *et al.*, 2012). Due to nonrandom sampling of *BRCA1* and *BRCA2* mutation carriers from different sites, a retrospective likelihood approach, developed by Antoniou *et al.* (2010) (Barnes *et al.*, 2012) and implemented in the ‘retrolike1.0.3’ program, was used to model the retrospective likelihood of the observed S346X A allele conditional on age at cancer diagnosis or age at last follow‐up. In this model, breast or ovarian cancer incidence was assumed to depend on the underlying genotype through a Cox proportional hazards model; the magnitude of association was estimated as a per‐allele log‐relative hazards ratio (HR) in a multiplicative model where each individual has either zero, one, or two copies of minor allele A for the S346X variant.

### 2.2 Plasmids, cell lines, and siRNA

The *Rad52^−/−^*mouse embryonic stem cell (mESC) cell line harboring the repeat‐mediated deletion (RMD) green fluorescent protein (GFP)‐based assay reporter (RMD‐GFP) was previously reported (Mendez‐Dorantes *et al.*, 2018). The *RAD52^KO^* human osteosarcoma U2OS cell line was previously reported (Kelso *et al.*, 2019) and was derived from the U2OS Flp‐In T‐Rex cell line (Zhou *et al.*, 2017). The plasmids for inducing DSBs in this reporter use single‐guide RNAs (sgRNAs) and Cas9, and are based on px330 (Ran *et al.*, 2013). The 5’268 and 9.1 kbp sgRNA/Cas9 plasmids, pCAGGS‐BSKX empty expression vector (EV), and pCAGGS‐NZE‐GFP were described previously (Bennardo *et al.*, 2008; Bennardo *et al.*, 2009; Bhargava *et al.*, 2017; Mendez‐Dorantes *et al.*, 2018). The pCAGGS‐hRAD52 complementation vector was generated by amplifying the *hRAD52* coding sequence from plasmid hRAD52‐GFP (from Simon Powell, Memorial Sloan Kettering Cancer Center) with the addition of a Kozak sequence, Flag tag, *Eco*RI site on the 5’ end, and an *Xho*I site on the 3′ end. The PCR product was digested and ligated into the *Eco*RI and *Xho*I sites of pCAGGS‐BSKX. The pCAGGS‐hRAD52 S346X plasmid was created by site‐directed mutagenesis using the Phusion Site‐Directed Mutagenesis Kit (Thermo Scientific, Waltham, MA USA). For depletion of BRCA2, we used either the nontargeting siRNA (siCTRL) (GE Dharmacon, Lafayette, CO, USA, D‐001810‐01) or a pool of four siRNAs of siBRCA2 (GE Dharmacon J‐042993‐05, J‐042993‐06, J‐042993‐07, and J‐042993‐08).

### 2.3 SSA DSB reporter assay

For the SSA reporter assay, 5.0 × 10^4^ RMD‐GFP *Rad52^−/−^* mESCs were plated per well in a 24‐well plate. To compare wild‐type (WT) hRAD52 and hRAD52 S346X, each well was transfected with 200 ng of 5’268 and 9.1 kbp sgRNA/Cas9 plasmids and 200 ng of either pCAGGS, pCAGGS‐hRAD52, or pCAGGS‐hRAD52 S346X using 1.8 µL of Lipofectamine 2000. For the siRNA analysis, transfections included 5 pmol of siCTRL or siBRCA2 siRNAs. Transfection was performed in 0.5 mL of antibiotic‐free media for 4 h, after which the transfection media was replaced with 2 mL media containing antibiotics. The percentage of GFP^+^ cells was quantified by flow cytometry 3 days after transfection on a CyAn Advanced Digital Processing Analyzer (Dako, Carpinteria, CA, USA). For each experiment, the frequency of GFP^+^ cells was normalized to transfection efficiency, as described previously (Bhargava *et al.*, 2018). Statistical analysis of the RMD reporter experiments was performed using the methods described in each figure legend. *P*‐values were adjusted for multiple comparisons.

### 2.4 Immunoblot analysis

To analyze cellular localization of hRAD52 and hRAD52 S346X, 2.0 × 10^5^ RMD‐GFP *Rad52^−/−^* mESCs were plated per well in a 6‐well plate. Each well was transfected with 800 ng of either pCAGGS, pCAGGS‐hRAD52, or pCAGGS‐hRAD52 S346X using 7.2 µL of Lipofectamine 2000. Two days after transfection, the cells were collected and lysed using NETN buffer (20 mm Tris at pH 8.0, 100 mm NaCl, 1 mm EDTA, 0.5% Igepal, 1.25 mm DTT, Roche protease inhibitor, Basel, Switzerland) with multiple freeze/thaw cycles. Total cellular protein was fractionated by SDS/PAGE on NuPAGE 4–12% gradient gels (Invitrogen, Carlsbad, CA USA) and electrotransferred onto PVDF membranes. The membranes were probed with horseradish peroxidase (HRP)‐conjugated Flag (Sigma, A8592, St.Louis, MO, USA), BRCA2 (Abcam, ab27976, Cambridge, UK), actin (Sigma, A2066), and HRP goat anti‐rabbit secondary antibody (Abcam, ab205718) as appropriate, followed by addition of enhanced chemiluminescence reagent (Thermo Scientific) to develop horseradish peroxidase signals.

### 2.5 Immunofluorescence analysis

To assess the subcellular localization of hRAD52 WT and hRAD52 S346X, 2.0 × 10^5^ *RAD52^KO^* U2OS cells (Kelso *et al.*, 2019) were plated per well in a 12‐well plate. Each well was transfected with 400 ng of either pCAGGS‐hRAD52 or pCAGGS‐hRAD52 S346X using 3.6 µL of Lipofectamine 2000. Twenty‐four hours after transfection, the cells were plated onto chamber slides and then incubated for an additional 24 h. The slides were fixed with 4% paraformaldehyde and treated with 0.1 m glycine and 0.5% Triton X‐100 prior to probing with an antibody against Flag (Sigma, F3165), secondary Alexa Fluor 488r goat anti‐mouse antibody (Invitrogen, A11029), and DAPI using VECTASHIELD Mounting Medium (Vector Laboratories H1500, Burlingame, CA, USA). Confocal microscopy images were acquired at 63X magnification using the Zeiss LSM 880 (Oberkochen, Germany) Confocal Microscope along with ZEN Black image acquisition software.

## 3 Results

### 3.1 Association of *RAD52* S346X with risk of breast or ovarian cancer in women carrying pathogenic *BRCA1* or *BRCA2* mutations

We tested the association of *RAD52* S346X with risk of developing breast or ovarian cancer in a large cohort of *BRCA1* and *BRCA2* mutation carriers. The *RAD52* S346X variant was genotyped on a custom Illumina array called OncoArray (Amos *et al.*, 2017). We observed good separation of clusters for each of the three genotypes (Fig. S1B), and this variant passed the series of genotyping quality control steps developed for the OncoArray project (Amos *et al.*, 2017). Of 15 679 *BRCA1* mutation carriers, 459 were heterozygous and 3 were homozygous variant carriers (MAF = 0.017), and of 10 979 *BRCA2* mutation carriers, 277 were heterozygous and 4 were homozygous variant carriers (MAF = 0.013). In assessing association of this variant with cancer, of the 15 679 *BRCA1* carriers, 7889 and 2369 carriers were affected with breast and ovarian cancers, respectively; of 10 979 *BRCA2* carriers, 5605 carriers and 2369 carriers were diagnosed with breast and ovarian cancers, respectively. *RAD52* S346X was highly significantly associated with reduced risk of breast cancer for *BRCA2* carriers (Table 1 and Table S1); each copy of the minor allele was estimated to confer a per‐allele HR of 0.69 (95% CI, 0.56 to 0.86, *P* = 0.0008). There also was evidence of association of *RAD52* S346X with reduced breast cancer risk for *BRCA1* carriers (Table 1 and Table S1). However, both the strength of association measured by *P*‐value of 0.02 and magnitude of association measured by per‐allele HR of 0.78 (95% CI of 0.64 to 0.97) were weaker compared to that observed for *BRCA2* carriers. For ovarian cancers, although the HRs were of similar magnitude as for breast cancers in *BRCA1* and *BRCA2* carriers, respectively, the HRs were not significant (*BRCA2* carriers, *P* = 0.10 and *BRCA1* carriers, *P* = 0.11) likely reflecting the smaller number of events (Table 1 and Table S1). In the BCAC samples of 122 977 cases and 105 974 controls, the association was not significant (*P*‐value = 0.13); there were fewer carriers in the cases than the controls (regression coefficient = −0.047) (Michailidou *et al.*, 2017).

**Table 1:** Association of *RAD52* S346X and risk of developing breast or ovarian cancer for *BRCA1* and *BRCA2* carriers. CI, confidence interval.

Gene	Cancer	Sample size	Number of cases	*P*‐value	HR^a^	95% CI
*BRCA1*	Breast	15 679	7889	0.02	0.78	0.64–0.97
*BRCA1*	Ovarian	15 679	2369	0.11	0.79	0.60–1.05
*BRCA2*	Breast	10 979	5605	0.0008	0.69	0.56–0.86
*BRCA2*	Ovarian	10 979	848	0.10	0.71	0.47–1.07

^a^ The magnitude of association was estimated as a per‐allele hazards ratio (HR) in a multiplicative model where each individual has either zero, one, or two copies of minor allele A for the S346X variant.

### 3.2 Effect of RAD52 S346X on SSA frequency

The protective effect of *RAD52* S346X in carriers of pathogenic variants of *BRCA1* and *BRCA2* may parallel the synthetic lethality observed upon simultaneous disruption of *BRCA1/2* and *hRAD52* (Feng *et al.*, 2011; Lok *et al.*, 2013). This synthetic lethality correlates with a synergistic reduction in the efficiency of DSB repair (Feng *et al.*, 2011; Lok *et al.*, 2013), suggesting that *RAD52* S346X may decrease DSB repair. To test whether RAD52 S346X confers a defect in DSB repair, we used a recently developed reporter system to examine repair of DSBs by SSA, a mechanism supported by RAD52 in mammalian cells (Mendez‐Dorantes *et al.*, 2018). This assay determines the frequency of repeat‐mediated deletion (RMD) in mouse embryonic stem cells (mESCs). Notably, mouse and human RAD52 proteins show substantial conservation (i.e., 69% identity and 80% similarity) (Muris *et al.*, 1994), suggesting that hRAD52 could support SSA in *Rad52^−/−^* mESCs.

RMD by SSA is initiated by two DSBs positioned between a pair of nontandem repeats on the same chromosome. The DSBs are created at defined genomic locations through the catalytic activity of Cas9 programmed with specific single‐guide RNAs (sgRNAs) (Ran *et al.*, 2013). The first DSB is introduced 268 bp downstream of the 5′ repeat, while the second DSB is introduced 9.1 kbp upstream of the 3′ repeat (Mendez‐Dorantes *et al.*, 2018) (Fig. 1B). To study the function of hRAD52 in SSA, we cotransfected *Rad52^−/−^* mESCs containing a chromosomally integrated RMD‐GFP reporter with an empty expression vector (EV) or a vector containing either *hRAD52* or *hRAD52* S346X, as well as the sgRNA/Cas9 plasmids. Repair of the DSBs by SSA deletes the chromosomal DNA lying between the breaks and generates an intact GFP expression unit and GFP^+^ cells that can be scored by flow cytometric analysis. The frequency of RMD was calculated from the percentage of GFP^+^ cells observed three days after transfection.

As reported previously, the frequency of RMD was near the level of detection in EV‐transfected *Rad52^−/−^* mESCs, consistent with SSA being dependent on RAD52 (Mendez‐Dorantes *et al.*, 2018). Importantly, transfection with the WT hRAD52 expression vector suppressed this defect to a similar degree (5.1% GFP^+^ cells) as expression of mouse RAD52, suggesting that human and mouse RAD52 possess similar capacities to support SSA in mESCs (Fig. 1D; Mendez‐Dorantes *et al.*, 2018). Conversely, transfection with the hRAD52 S346X expression vector only partially suppressed the SSA defect, displaying RMD frequencies that were greater than twofold lower than in cells transfected with the WT hRAD52 expression plasmid (1.8% GFP^+^ cells, *P* < 0.0001; Fig. 1D). These findings suggest that hRAD52 S346X has a diminished capacity to promote SSA in mESCs. Immunoblot analysis indicated that similar levels of expression of the FLAG‐tagged WT and S346X proteins were expressed in these cells (Fig. 1C), suggesting that the differences in RMD frequency are unlikely to be due to different levels of WT and mutant hRAD52.

### 3.3 Depletion of BRCA2 causes an increase in RAD52‐dependent SSA

Because *hRAD52* S346X was observed to exert a protective effect in carriers of pathogenic *BRCA2* mutations (Table 1) and to diminish DSB repair (Fig. 1D), we examined how the interaction between the depletion of BRCA2 and expression of *hRAD52* S346X affected DSB repair by SSA. Expressing siRNAs that depleted levels of BRCA2 in *Rad52^−/−^* mESCs (Fig. 1E) resulted in similarly increased frequencies of RMD in cells expressing either hRAD52 (adjusted *P = *0.0043) or hRAD52 S346X (adjusted *P = *0.0172) (Fig. 1F), indicating that BRCA2 exerts a suppressive effect on SSA that is consistent with previous findings (Stark *et al.*, 2004). Further, the similar levels of stimulation observed upon depleting levels of BRCA2 in cells expressing WT and mutant hRAD52 indicate that the roles of BRCA2 and hRAD52 in determining levels of SSA are independent of one another. Additionally, depleting BRCA2 in conditions where hRAD52 S346X is expressed resulted in frequencies of SSA that were not significantly different than when hRAD52 is expressed without depletion of BRCA2 (siCTRL), suggesting that SSA would not become elevated upon inactivation of BRCA2 in the cells of *hRAD52* S346X carriers.

### 3.4 RAD52 S346X is predominantly localized in the cytoplasm

**Fig. 2:**
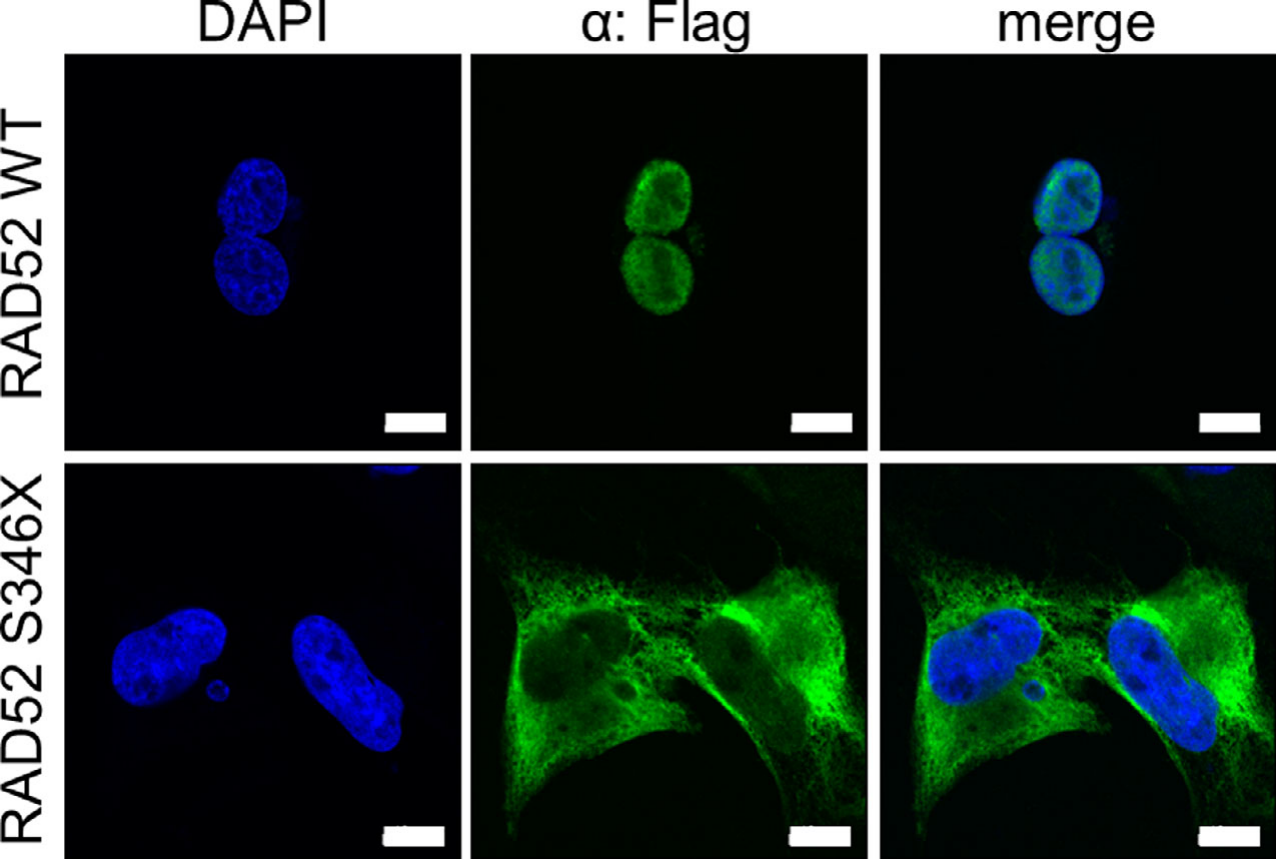
Subcellular localization of the WT and S346X RAD52 proteins tagged with Flag. *RAD52^KO^* cells transiently transfected with pCAGGS‐RAD52 WT or pCAGGS‐RAD52 S346X expression vectors were examined by immunofluorescence analysis with anti‐Flag antibody (green). Nuclei were stained with DAPI (blue). White scale bars, 10 µm.

Because hRAD52 S346X is missing the C‐terminal nuclear localization sequence (NLS) of WT hRAD52 (Koike *et al.*, 2013), we performed immunfluorescent staining and confocal microscopy to investigate the subcellular localization of the truncated protein. The *Rad52^KO^* U2OS human osteosarcoma cell line (Kelso *et al.*, 2019) was transfected with expression vectors for WT or S346X hRAD52 tagged with a Flag epitope. Immunofluorescence analysis using anti‐Flag antibody showed that hRAD52 WT tagged with Flag localized to the nucleus, while hRAD52 S346X tagged with Flag predominantly localized to the cytoplasm (Fig. 2).

## 4 Discussion

RAD52 is a protein that can mediate the annealing of complementary strands of DNA (Rothenberg *et al.*, 2008; Symington, 2002) and has been shown to be important for repair of DSBs by SSA, an obligatorily mutagenic repair mechanism (Mendez‐Dorantes *et al.*, 2018; Morales *et al.*, 2015; Stark *et al.*, 2004; Symington, 2002). *hRAD52* S346X is a mutation that codes for a RAD52 protein with 17.2% of its amino acid sequence absent from its C terminus (Fig. 1A). We found that this mutation protected against the development of breast cancer in *BRCA1/2* mutation carriers (Table 1). We further showed that *hRAD52* S346X conferred a significantly reduced frequency of DSB repair by SSA in *Rad52^−/−^* mESCs compared to *hRAD52*. In contrast, attenuating levels of BRCA2 elevated the frequency of SSA, perhaps due to a reduction in the mediation of RAD51 recombinase function, which suppresses SSA by supporting HDR, a competitive mechanism of DSB repair that conserves genome structure (Bhargava *et al.*, 2016). Importantly, *hRAD52* S346X suppressed the elevated frequency of SSA caused by reducing the level of BRCA2. This suggests that *hRAD52* S346X may suppress tumorigenesis in BRCA2‐deficient cells by suppressing the mutagenic effects of elevated SSA. Alternatively, the suppression of SSA by *hRAD52* S346X may block tumor formation in BRCA2‐deficient cells because of their loss of two mechanisms of DSB repair, leading to the increased persistence of DSBs and apoptosis.

The last eight amino acids of mammalian RAD52 function as a nuclear localization sequence (NLS) (Koike *et al.*, 2013). We hypothesized that the reduced DSB repair by SSA observed when *hRAD52* S346X is expressed in *Rad52^−/−^* mESCs as compared to *hRAD52* may be due to a lack of hRAD52 protein in the nucleus. Through immunostaining and confocal microscopy, we determined that hRAD52 S346X protein is predominantly localized to the cytoplasm. Therefore, we consider it likely that the reduction in nuclear hRAD52 S346X protein is responsible for the decreased repair of DSBs via SSA. Supporting our hypothesis, it has been shown that the protein encoded by the germline *NEIL1* Q282X variant also lacks a C‐terminal NLS, resulting in reduced nuclear localization of the protein (Shinmura *et al.*, 2015). The NEIL1 DNA glycosylase is involved in repair of oxidized bases via the base excision repair pathway, and the Q282X variant displays a diminished ability to suppress mutations (Shinmura *et al.*, 2015). Since hRAD52 S346X still has all the other known functional domains (Fig. 1), it likely retains similar capabilities as that of the full‐length protein once in the nucleus. For example, hRAD52 S346X likely retains DNA‐binding capabilities based on previous findings that truncated hRAD52 peptides are as effective in strand annealing as that of full‐length hRAD52 (Kagawa *et al.*, 2001; Ranatunga *et al.*, 2001). Still, we acknowledge the possibility that the C‐terminal amino acids missing from hRAD52 S346X could play an as yet unknown role in SSA.

The Powell group made the important findings that reducing the cellular levels of RAD52 leads to synthetic lethality when levels of the HDR factors BRCA2, BRCA1, and PALB2, and the RAD51 paralogs are also reduced (Chun *et al.*, 2013; Feng *et al.*, 2011; Lok *et al.*, 2013). The findings of Powell and others suggest that DSB repair by RAD52‐dependent HDR and/or SSA may act as backups to DSB repair by the canonical, BRCA‐mediated mechanism of HDR (Feng *et al.*, 2011; Stark *et al.*, 2004; Wray *et al.*, 2008). Our results corroborate these findings, as we observed that the *hRAD52* truncation variant is protective against development of cancer in women carrying pathogenic *BRCA2* mutations and that this mutation confers a decrease in DSB repair by SSA.

We speculate that our observation that *hRAD52* S346X confers both a loss of DSB repair and the protection of *BRCA2^‐^*mutation carriers against breast cancer will impact future cancer treatments. For instance, tumor cells from carriers of both pathogenic *BRCA2* mutations and *hRAD52* S346X should have a reduced ability to survive when exposed to chemotherapy‐induced DSBs. Recently, there has been a concerted effort to identify small molecular inhibitors of hRAD52 in the hope of clinically treating BRCA‐deficient breast and ovarian cancers (Chandramouly *et al.*, 2015; Huang *et al.*, 2016; Sullivan *et al.*, 2016). Although no clinical trials have been reported for human cancers, a recent study showed that an inhibitor of RAD52 could reduce the growth of BRCA1‐deficient tumors in mice (Sullivan‐Reed *et al.*, 2018). Interestingly, when these mice were treated with a combination of both RAD52 and PARP inhibitors, tumor growth was inhibited completely, suggesting a synergistic effect (Sullivan‐Reed *et al.*, 2018). This finding is of particular interest due to the common phenomenon of acquired resistance to PARP inhibitors by BRCA‐deficient tumors (Lord and Ashworth, 2013). Ideally, future treatment of HDR‐deficient cancers will employ the synergistic effect of combining inhibitors of multiple DNA repair pathways. Further, evaluating the status of *hRAD52* in BRCA‐deficient tumors may be advised as tumors that are defective for both hRAD52 and BRCA2 could show a more lasting response to PARP‐inhibitor treatment.

The physiological relevance of reduced SSA activity could affect other RAD52‐dependent events apart from the repair of DSBs by SSA per se. In addition to the repair of DSBs via SSA and HDR, hRAD52 has been implicated in break‐induced replication (BIR) (Sotiriou *et al.*, 2016), transcription‐coupled homologous recombination (Teng *et al.*, 2018), processing of stalled replication forks (Malacaria *et al.*, 2019), mitotic DNA synthesis (MiDAS) (Bhowmick *et al.*, 2016), and alternative lengthening of telomeres (Zhang *et al.*, 2019), as well as several other pathways (Jalan *et al.*, 2019). Thus, hRAD52 likely plays an important role in maintaining the viability of cancer cells under replication stress and could help explain the protective effect of *hRAD52* S346X. Because replication stress is so prominent in cancer, it is possible that treatment with RAD52 inhibitors will have therapeutic effect even in HDR‐proficient tumor cells.

## 5 Conclusion

This is the first study to report the protective effect of a germline coding variant, *hRAD52* S346X in individuals carrying germline pathogenic variants in the homologous recombination gene, BRCA2. This *RAD52* mutation causes a reduction in DSB repair by SSA. There likely are additional variants in other DNA repair genes that by themselves do not significantly alter risk of developing cancer, but have profound effects in combination with a pathogenic variant in a second gene also involved in DNA repair.

## Conflict of interest

The authors declare no conflict of interest.

## Author contributions

SNL and YCD conceived the project. SNL and JMS designed the project. AWA performed the experiments. YCD, AWA, and CMD analyzed the data. AWA, YCD, AMB, JMS, and SNL wrote and edited the manuscript. All authors approved the final manuscript.

## Supporting information


**Fig. S1.** (A) Initial S346X mutation detection by Sanger sequencing; (B) Clustering of the S346X genotypes from the Illumina Oncoarray.

**Table S1.** Sample distribution by gene, cancer, and RAD52 S346X genotypes.
