# Supplementary figures and images for: The RAD52 S346X variant reduces risk of developing breast cancer in carriers of pathogenic germline BRCA2 mutations
Source: Mol Oncol. 2020 Apr 25;14(6):1124–33. doi: 10.1002/1878-0261.12665 (PMC7266271; doi:10.1002/1878-0261.12665)

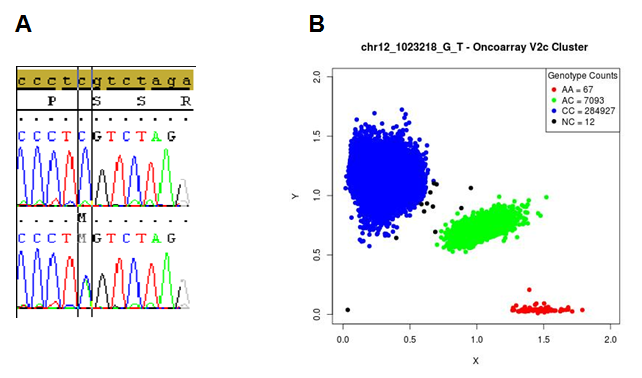

Supplement: Supplementary file 1 — Fig. S1. (A) Initial S346X mutation detection by Sanger sequencing; (B) Clustering of the S346X genotypes from the Illumina Oncoarray. [file MOL2-14-1124-s001.tif]
