# Supplementary material for: The RAD52 S346X variant reduces risk of developing breast cancer in carriers of pathogenic germline BRCA2 mutations
Source: Mol Oncol. 2020 Apr 25;14(6):1124–33. doi: 10.1002/1878-0261.12665 (PMC7266271; doi:10.1002/1878-0261.12665)
Supplement: Supplementary file 2 — Table S1. Sample distribution by gene, cancer, and RAD52 S346X genotypes. [file MOL2-14-1124-s002.docx]

Supplemental Table 1: Sample distribution by gene, cancer, and *RAD52* S346X genotypes

| Gene | | *BRCA1* carriers (n = 15679) | | | | *BRCA2* carriers (n = 10979) | | | |
| --- | --- | --- | --- | --- | --- | --- | --- | --- | --- |
| Cancer type | | Breast cancer | | Ovarian Cancer | | Breast cancer | | Ovarian Cancer | |
| Cancer status | | no | yes | no | yes | no | yes | no | yes |
| RAD52 S346X genotype | C/C | 7567 | 7650 | 12911 | 2306 | 5212 | 5486 | 9877 | 821 |
|  | C/A | 221 | 238 | 396 | 63 | 159 | 118 | 250 | 27 |
|  | A/A | 2 | 1 | 3 | 0 | 3 | 1 | 4 | 0 |
